# Supplementary material for: Structural basis for botulinum neurotoxin E recognition of synaptic vesicle protein 2
Source: Nat Commun. 2023 Apr 24;14:2338. doi: 10.1038/s41467-023-37860-8 (PMC10125960; doi:10.1038/s41467-023-37860-8)
Supplement: Supplementary file 1 — Supplementary Information [file 41467_2023_37860_MOESM1_ESM.pdf]

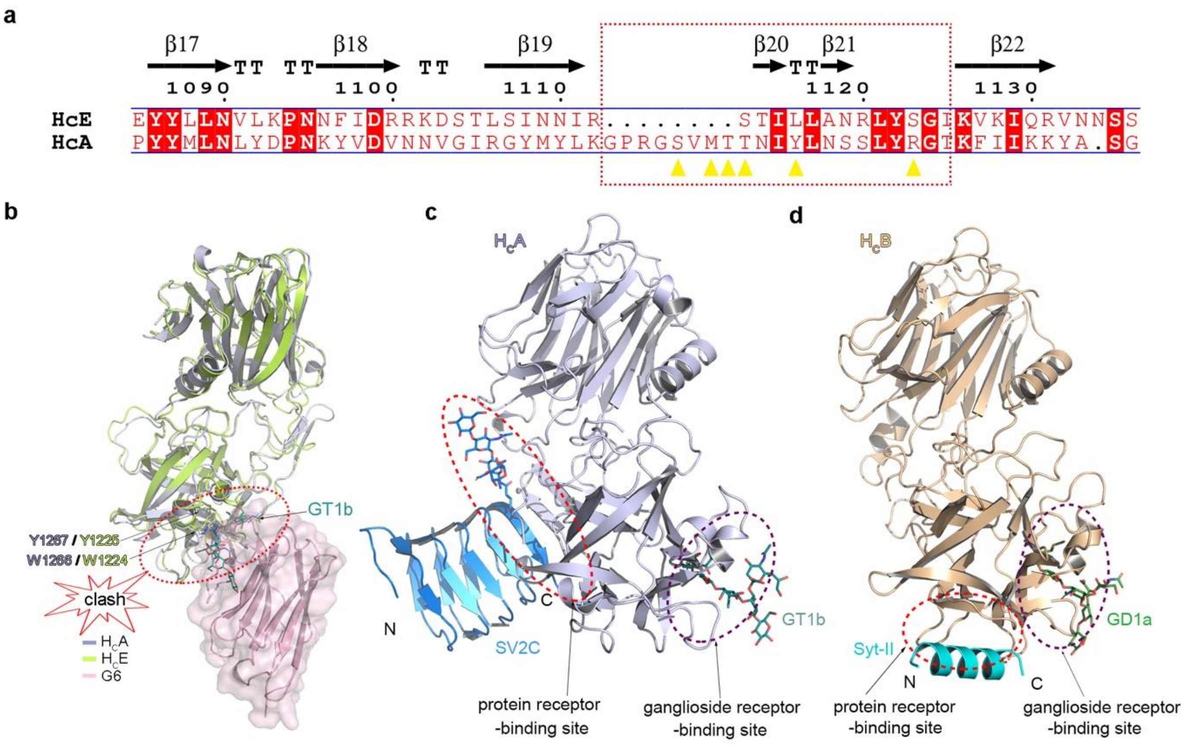

Supplementary Figure 1. Comparing the receptor-binding modes among H_CA, H_CB, and H_CE. a, Amino acid sequence alignment between H_CA and H_CE (prepared using MultAlin [1] and ESPript 3.0 [2]) focusing on the major area where H_CA recognizes the protein moiety of SV2C. Identical residues are indicated with white letters on a red background and similar conserved residues are in red letters. The residues of H_CA that bind to SV2C protein moiety are indicated by yellow triangles, and the major differences between H_CA and H_CE are highlighted in a red box. Residue numbers and the secondary structures of H_CE are shown on the top. **b**, G6 occupies the ganglioside-binding pocket on H_CE based on comparison of the H_CE–G6 complex and the H_CA–GT1b complex (PDB: 2VU9 [https://www.rcsb.org/structure/2VU9]): H_CA (blue white cartoon); GT1b (deep teal sticks); H_CE (lemon cartoon); G6 (pink surface and cartoon). Residues "WY" of the highly conserved "SxWY" motif are shown as sticks. **c**, A model of H_CA in complex with SV2C and GT1b based on the structures of the H_CA–SV2C (PDB: 5JLV [https://www.rcsb.org/structure/5JLV]) and the H_CA–GT1b (PDB: 2VU9 [https://www.rcsb.org/structure/2VU9]) complexes: H_CA (blue white); SV2C (blue); GT1b (deep teal sticks). **d**, H_CB (wheat) in complex with Syt-II (cyan) and GD1a (green sticks) (PDB: 4KBB [https://www.rcsb.org/structure/4KBB]).

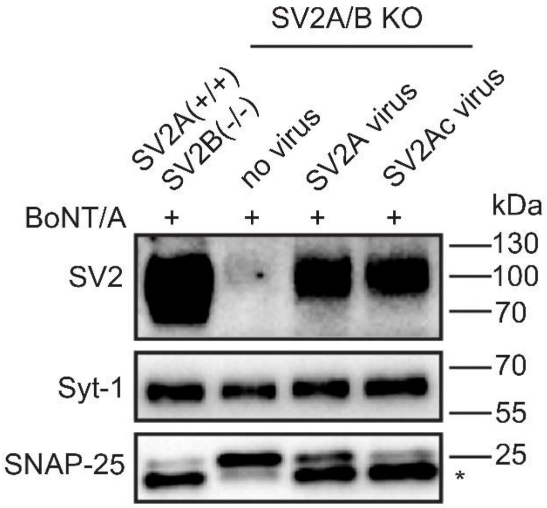

Supplementary Figure 2. SV2Ac mediates BoNT/A entry into cultured cortical neurons. The full-length SV2A and SV2Ac were expressed via lentiviral transduction in cortical neurons cultured from SV2A/B double KO mice. Neurons were exposed BoNT/A (100 pM, 24-hour incubation in medium). Cell lysates were collected and analyzed by immunoblot. The SNAP-25 antibody can detect both the intact SNAP-25 and the smaller fragment of SNAP-25 after cleavage by BoNT/A (marked with *). Syt-1 was detected as a loading control. SV2A(+/+)/SV2B(-/-) neurons were tested in parallel as a positive control. A representative result is shown (n=2). Source data are provided as a Source Data file.

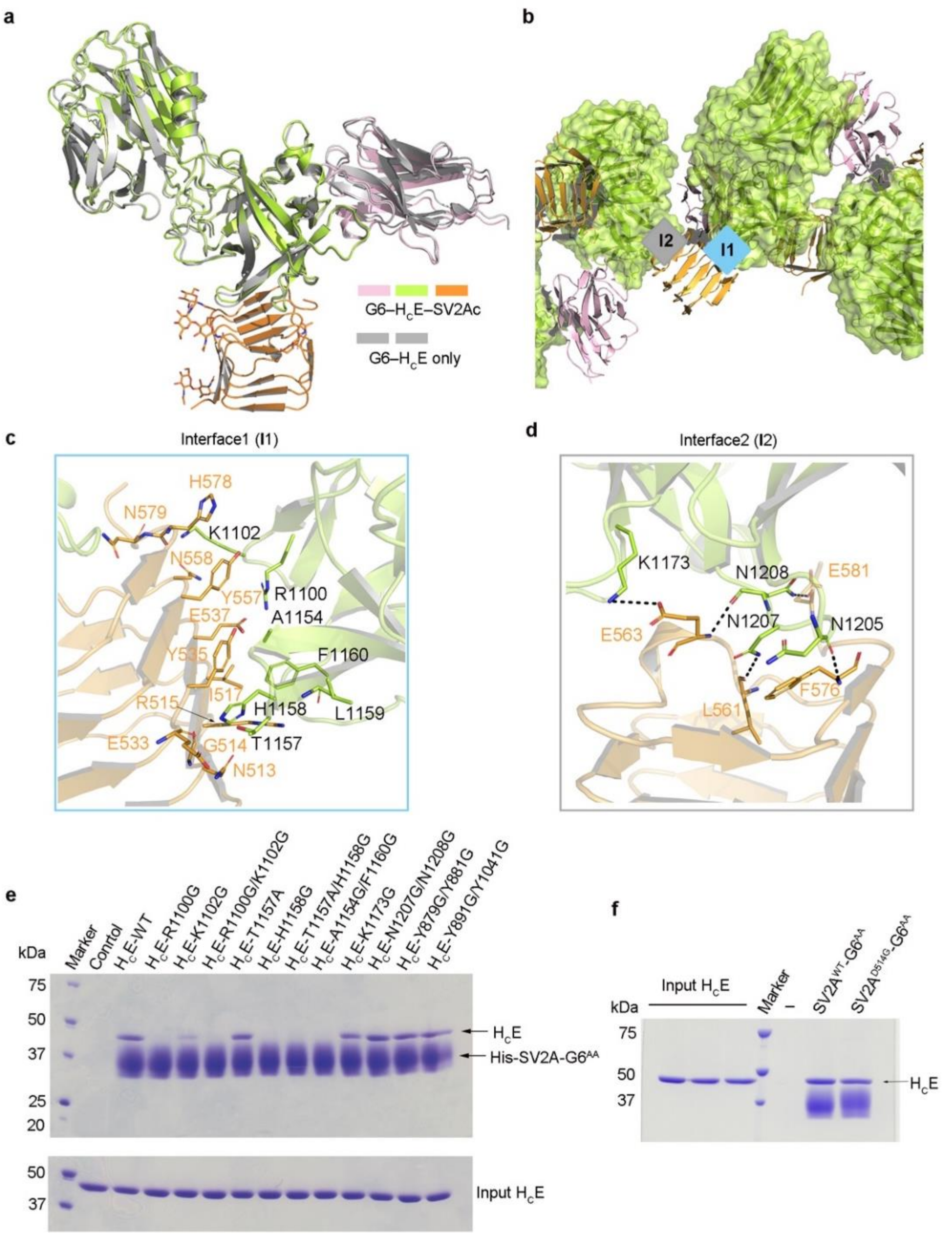

Supplementary Figure 3. Structure of the H$_C$E–SV2Ac–G6 complex and mutagenesis studies on H$_C$E. **a**, The structure of the H$_C$E–SV2Ac–G6 complex (H$_C$E, lemon; G6, pink; SV2Ac, orange) is superimposed with the H$_C$E–G6 complex (grey) based on H$_C$E. It shows that the SV2Ac-linked G6 and the stand alone G6 bind to H$_C$E in an identical manner. **b**, Two different interfaces were observed between SV2Ac and H$_C$E in the crystal of the H$_C$E–SV2Ac–G6 complex (I1 and I2). **c-d**, The interacting residues of SV2Ac and H$_C$E at I1 and I2 are shown as sticks. **e**, Characterization of the interactions between H$_C$E and SV2A by structure-based mutagenesis. Pull-down assays were performed using H$_C$E variants as preys and the His-tagged SV2A–G6^{AA} as a bait. **f**, SV2A^{D514G} (D514 of SV2A was replaced with the equivalent G500 of SV2C) and SV2A^{WT} bind to H$_C$E with similar affinities based on a pull-down assay with H$_C$E as a prey and the equal amount of His-tagged SV2A^{D514G}–G6^{AA} and SV2A^{WT}–G6^{AA} fusion proteins as baits. Representative results are shown in panels **e** and **f** (n=3).

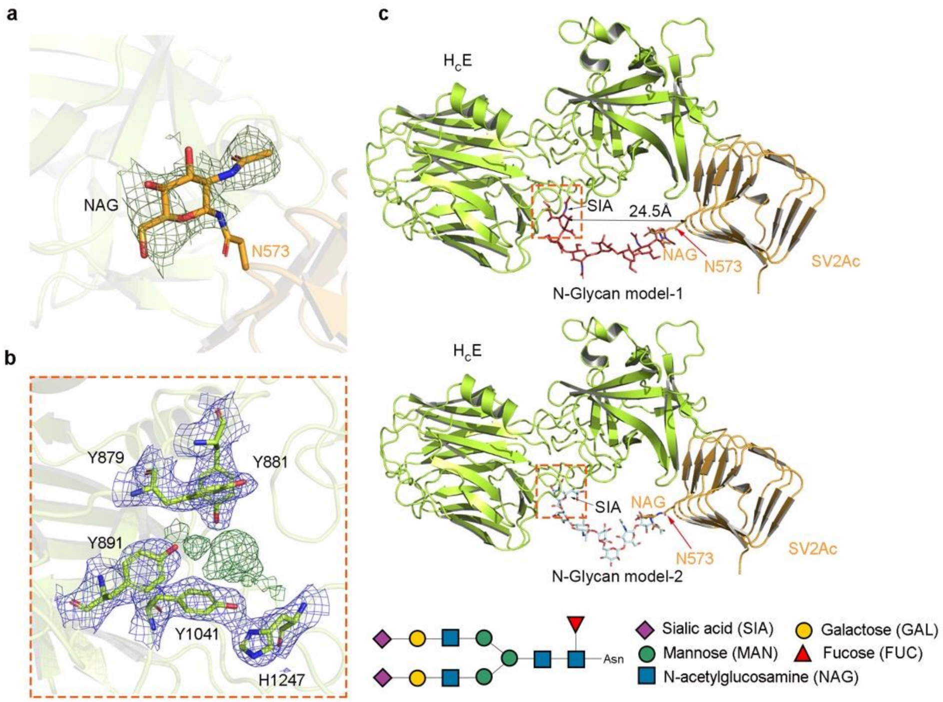

Supplementary Figure 4. BoNT/E grips the distant tip of SV2A glycan. a, The electron density of N-acetylglucosamines (NAG) of the SV2Ac-N573 glycan observed in the H_CE–SV2Ac–G6 complex. An omit electron density map contoured at 1.5 σ was overlaid with the final refined model. **b**, Electron densities for an unknown molecule were observed in a pocket located at the N-terminal sub-domain of H_CE ($H_{CN}E$). An omit electron density map contoured at 1.5 σ (green) and a $2F_O$-F_C electron density map for five selected residues (sticks) in this area contoured at 1 σ (blue) were overlaid with the final refined model. **c**, Structure modeling of H_CE binding to the SV2A N573 glycan. Two representative complex-type N-glycans are modeled to represent the SV2A-N573 glycan based on the structures of a glycan from human prostate-specific antigen (upper panel, red sticks, PDB: 3QUM [https://www.rcsb.org/structure/3QUM]) and a sialylated human IgG Fc (lower panel, pale cyan sticks, PDB: 4BYH [https://www.rcsb.org/structure/4BYH]). The terminal sialic acid of both glycans could reach the sialic acid-binding pocket on H_CE (orange dotted area). A schematic representation of a complex-type N-glycan structures is shown at the bottom.

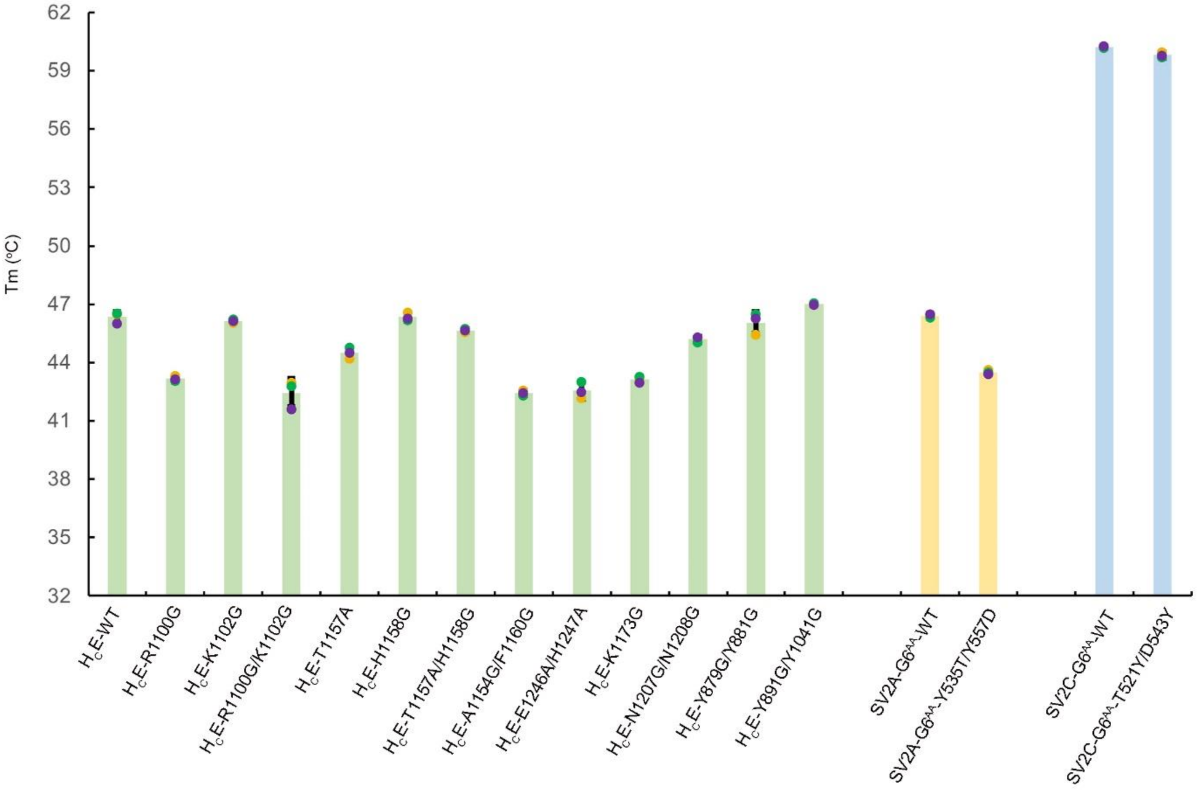

Supplementary Figure 5. H_CE, SV2A–G6^{AA} and SV2C–G6^{AA} variants adopt wild-type-like structures. The thermal stability of proteins was measured using a fluorescence-based thermal shift assay on a StepOne real-time PCR system (ThermoFisher). Protein melting was monitored using a hydrophobic dye, SYPRO Orange (Sigma-Aldrich), as the temperature was increased in a linear ramp from 25°C to 95°C. The midpoint of the protein-melting curve (Tm) was determined using the software provided by the instrument manufacturer. The data are presented as means ± s.d. (n=3). All the H_CE, SV2A–G6^{AA} and SV2C–G6^{AA} variants showed Tm values comparable to the wild-type proteins, indicating correct protein folding.

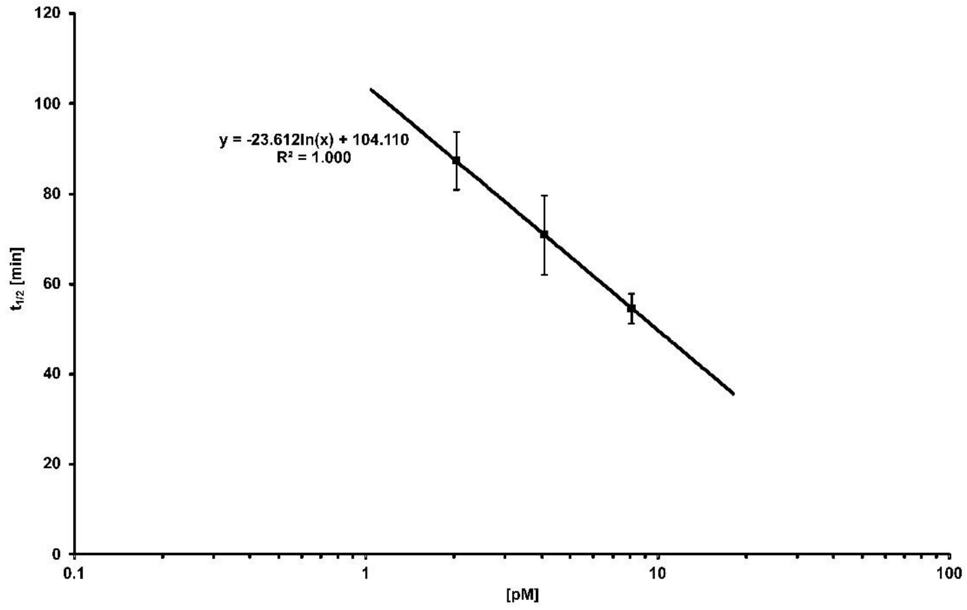

Supplementary Figure 6. Dose-response curve of the wild-type BoNT/E1 in the mouse phrenic nerve hemidiaphragm assay. To allow comparison of the altered neurotoxicity of mutants with BoNT/E1 wild-type, BoNT/E1 wild-type was tested in three concentrations (2.0 pM, n = 6; 4.0 pM, n = 8; 8.0 pM, n = 5 biologically independent experiments). A logarithmic function (y(BoNT/E1 wt; 2.0, 4.0, 8.0 pM) = -23.61 ln(x) + 104.11, R2 = 0.999) was fitted to the data points (mean ± SD) and used to convert paralytic-halftimes of BoNT/E1 mutants into concentrations of the wild-type and finally expressed as relative neurotoxicity.

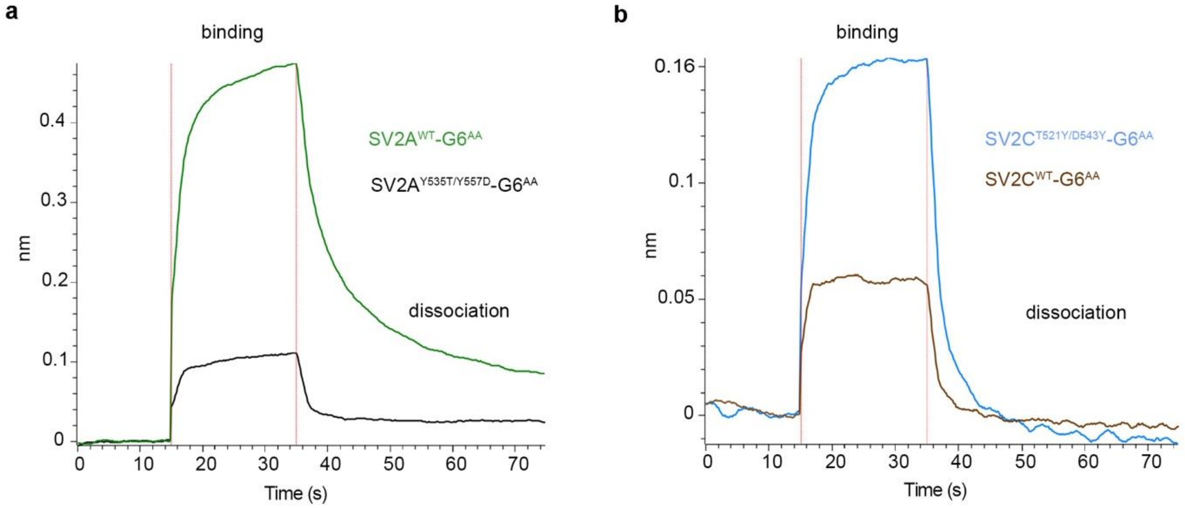

Supplementary Figure 7. Bio-layer interferometry (BLI) analyses of SV2A–G6^{AA} and SV2C–G6^{AA} variants binding to H$_{C}$E. a, Equal amounts of biotinylated SV2A–G6^{AA} or SV2A$^{Y535T/Y557D}$–G6^{AA} (400 nM) were immobilized on streptavidin (SA) biosensors as ligands and 1 µM of H$_{C}$E was used as the analyte. **b**, Equal amounts of biotinylated SV2C–G6^{AA} or SV2C$^{T521Y/D543Y}$–G6^{AA} were immobilized on streptavidin (SA) biosensors as ligands and 1 µM of H$_{C}$E was used as the analyte.

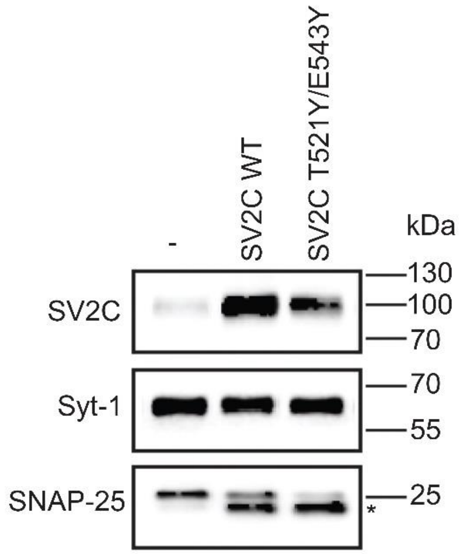

Supplementary Figure 8. SV2C(T521Y/E543Y) mediates BoNT/A entry into cultured neurons. The full-length SV2C and SV2C(T521Y/E543Y) were expressed via lentiviral transduction in cortical neurons cultured from SV2A/B KO mice. Neurons were exposed to BoNT/A (1 nM, 12 hours in medium). Cell lysates were harvested and analyzed by immunoblot assays. Cleaved SNAP-25 is marked with *. Expression of SV2C is detected using a polyclonal anti-SV2C antibody. Both SV2C WT and SV2C(T521Y/E543Y) mediated entry of BoNT/A, resulting in cleavage of SNAP-25. A representative result is shown (n=2). Source data are provided as a Source Data file.

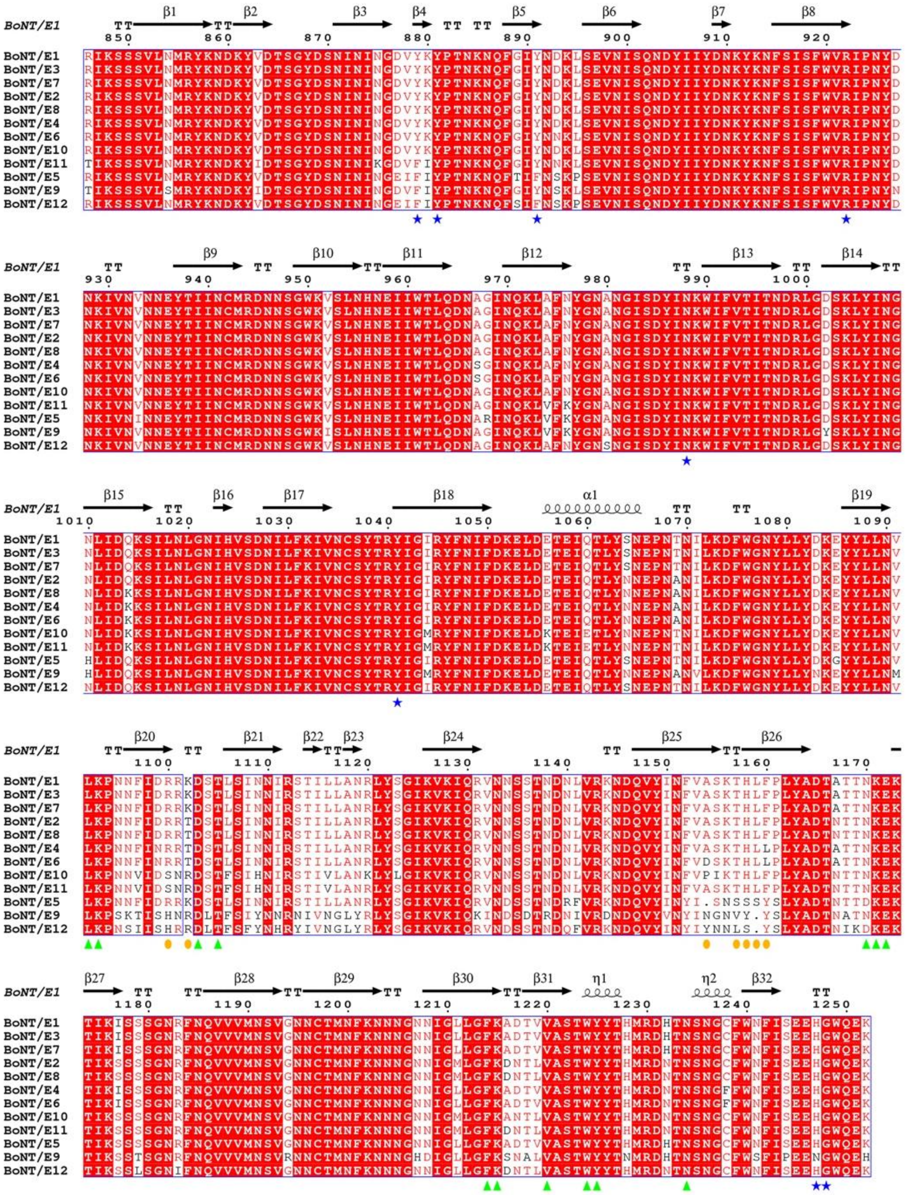

Supplementary Figure 9. Sequence alignment among twelve BoNT/E subtypes. The amino acid sequences of BoNT/E1-E12 are taken from GenBank: AFV91350 (E1), EF028404 (E2), ABM73980 (E3), EEP52948 (E4), AB037704 (E5), A8Y878 (E6), AER11391 (E7), AER11392 (E8), AFV91339 (E9), AII82300 (E10), KF861879 (E11), and KF929215 (E12). Key H_CE residues that are recognized by VHH G6 are indicated by green triangles. H_CE residues that interact with SV2Ac peptide or sialic acid are labeled by orange ovals or blue stars, respectively.

Supplementary Table 1. Data collection, phasing and refinement statistics.

	H_CE–G6 (PDB: 7UIE)	H_CE–SV2Ac–G6 (PDB: 7UIA)	H_CE–SV2Ac–G6 (soaking SIA) (PDB: 7UIB)
**Data collection**			
Space group	P 21 21 21	C 2 2 21	C 2 2 21
Cell dimensions			
a, b, c (Å)	88.74, 174.63, 214.44	140.74, 172.34, 137.16	141.37, 172.18, 137.05
α, β, γ (°)	90.00, 90.00, 90.00	90.00, 90.00, 90.00	90.00, 90.00, 90.00
Wavelength (Å)	0.97918	0.97918	0.97918
Resolution (Å)	174.63 - 3.23 (3.32 - 3.23) *	137.16 - 2.59 (2.67 - 2.59) *	137.05 - 2.77 (2.87 - 2.77) *
R_{merge} (%)	22.4 (>100)	9.8 (>100)	9.0 (55.8)
$I/\sigma I$	8.6 (1.0)	19.9 (2.6)	14.6 (3.2)
$CC_{1/2}$	0.987 (0.333)	0.999 (0.894)	0.993 (0.900)
Completeness (%)	99.6 (99.7)	100 (100)	99.8 (99.9)
Redundancy	4.1 (4.2)	12.1 (12.5)	6.2 (6.8)
**Refinement**			
Resolution (Å)	135.41 - 3.23 (3.32 - 3.23) *	109.01 - 2.59 (2.67 - 2.59) *	109.26 - 2.77 (2.87 - 2.77) *
No. reflections	53969	52103	42703
R_{work} / R_{free}	23.0 / 27.3	22.5 / 25.0	23.5 / 26.1
No. atoms			
Protein	21293	10169	10189
Ligand/ion	0	182	202
Water	0	159	178
B-factors			
Protein	91.95	57.21	55.52
Ligand/ion	-	80.87	80.78
Water	-	43.24	44.03
Ramachandran plot			
Favored (%)	95.15	94.6	95.4
Allowed (%)	4.85	5.4	4.6
Outliers (%)	0	0	0
R.m.s deviations			
Bond lengths (Å)	0.002	0.005	0.003
Bond angles (°)	0.48	1.138	0.814

*Values in parentheses are for highest-resolution shell.
The dataset was derived from a single crystal.

Supplementary Table 2. Protein-protein interactions in the BoNT/E–SV2Ac complex.

The amino acids on BoNT/A that are structurally equivalent to the SV2A-binding residues on BoNT/E are listed for comparison, while the BoNT/E-interacting residues on human SV2A and SV2B, and the equivalent residues on SV2C are also listed. The conserved residues are colored black, the homologous substitutions are in green, while non-homologous replacements are in red. The residues colored in light blue are involved in main-chain-mediated interactions.

BoNT/A	BoNT/E	Type of interaction	SV2Ac	SV2A	SV2B	SV2C
E1190	T1157	HB	N513	N513	N456	N499
Y1191	H1158	HB	G514 (mc)	D514	D457	G500
R1192	L1159 (mc)	HB	R515	K515	K458	R501
L1193	F1160	vdW	I517	I517	T460	I503
E1190	T1157	HB	E533 (mc)	E533	E476	S519
L1193 / K1187	A1154 / F1160	vdW	Y535	Y535	Y478	T521
V1125	R1100	SB	E537	E537	E480	E523
		HB	Y557	Y557	Y500	D543
N1127	K1102	HB	N558	N558	N501	N544
		HB	H578 (mc)	H578	E521	H564
		HB	N579 (mc)	N579	Q522	N565

“SB”, “vdW”, and “HB” stand for salt bridge, van der Waals interaction, and hydrogen bond, respectively. “mc” indicates the main-chain-mediated contacts, and all the other contacts are mediated by side-chain atoms.

Supplementary References

1. Corpet, F. Multiple sequence alignment with hierarchical clustering. *Nucleic Acids Res* **16**, 10881-90 (1988).
2. Robert, X. & Gouet, P. Deciphering key features in protein structures with the new ENDscript server. *Nucleic Acids Res* **42**, W320-4 (2014).
