# Supplementary material for: Structural basis for botulinum neurotoxin E recognition of synaptic vesicle protein 2
Source: Nat Commun. 2023 Apr 24;14:2338. doi: 10.1038/s41467-023-37860-8 (PMC10125960; doi:10.1038/s41467-023-37860-8)
Supplement: Supplementary file 3 — Reporting Summary [file 41467_2023_37860_MOESM3_ESM.pdf]

nature portfolio

Corresponding author(s): Rongsheng Jin & Min Dong

Last updated by author(s): Mar 23, 2023

Reporting Summary

Nature Portfolio wishes to improve the reproducibility of the work that we publish. This form provides structure for consistency and transparency in reporting. For further information on Nature Portfolio policies, see our Editorial Policies and the Editorial Policy Checklist.

Statistics

For all statistical analyses, confirm that the following items are present in the figure legend, table legend, main text, or Methods section.

n/a	Confirmed	
☐	☒	The exact sample size (*n*) for each experimental group/condition, given as a discrete number and unit of measurement
☐	☒	A statement on whether measurements were taken from distinct samples or whether the same sample was measured repeatedly
☒	☐	The statistical test(s) used AND whether they are one- or two-sided<br>*Only common tests should be described solely by name; describe more complex techniques in the Methods section.*
☒	☐	A description of all covariates tested
☒	☐	A description of any assumptions or corrections, such as tests of normality and adjustment for multiple comparisons
☐	☒	A full description of the statistical parameters including central tendency (e.g. means) or other basic estimates (e.g. regression coefficient) AND variation (e.g. standard deviation) or associated estimates of uncertainty (e.g. confidence intervals)
☒	☐	For null hypothesis testing, the test statistic (e.g. *F*, *t*, *r*) with confidence intervals, effect sizes, degrees of freedom and *P* value noted<br>*Give P values as exact values whenever suitable.*
☒	☐	For Bayesian analysis, information on the choice of priors and Markov chain Monte Carlo settings
☒	☐	For hierarchical and complex designs, identification of the appropriate level for tests and full reporting of outcomes
☒	☐	Estimates of effect sizes (e.g. Cohen's *d*, Pearson's *r*), indicating how they were calculated

Our web collection on statistics for biologists contains articles on many of the points above.

Software and code

Policy information about availability of computer code

Data collection	UNICORN 5.2 control and analysis software (Cytiva); VitroDat Online software (v3.4)
Data analysis	GraphPad Prism 7; UNICORN 5.2 control and analysis software (Cytiva); the X-ray diffraction data were processed with XDS as implemented in RAPD v2.0.1 (https://github.com/RAPD/RAPD); Coot v.0.8.9.2 and Phenix v1.18.2 were used to build, refine and evaluate all the atomic models; ForteBio Data analysis HT 10.0 was used to process BLI data; Pymol 2.0.7 were used to generate figures

For manuscripts utilizing custom algorithms or software that are central to the research but not yet described in published literature, software must be made available to editors and reviewers. We strongly encourage code deposition in a community repository (e.g. GitHub). See the Nature Portfolio guidelines for submitting code & software for further information.

Data

Policy information about availability of data

All manuscripts must include a data availability statement. This statement should provide the following information, where applicable:

- Accession codes, unique identifiers, or web links for publicly available datasets
- A description of any restrictions on data availability
- For clinical datasets or third party data, please ensure that the statement adheres to our policy

All data generated or analyzed during this study are included in this article or its supplementary information.

Human research participants

Policy information about studies involving human research participants and Sex and Gender in Research.

Reporting on sex and gender	N/A
Population characteristics	N/A
Recruitment	N/A
Ethics oversight	N/A

Note that full information on the approval of the study protocol must also be provided in the manuscript.

Field-specific reporting

Please select the one below that is the best fit for your research. If you are not sure, read the appropriate sections before making your selection.

☒ Life sciences ☐ Behavioural & social sciences ☐ Ecological, evolutionary & environmental sciences

For a reference copy of the document with all sections, see nature.com/documents/nr-reporting-summary-flat.pdf

Life sciences study design

All studies must disclose on these points even when the disclosure is negative.

Sample size	For statistical analysis of neuron-based immunoblots, 2 to 3 different doses were tested for each experimental group.
Data exclusions	No data are excluded from analysis
Replication	Immunoblots have been replicated at least 2 times.<br>For statistical analysis, immunoblots have been replicated at least 3 times.
Randomization	The experiments did not require randomization.
Blinding	Our experiments did not include subjective measurements and did not require blinding.

Reporting for specific materials, systems and methods

We require information from authors about some types of materials, experimental systems and methods used in many studies. Here, indicate whether each material, system or method listed is relevant to your study. If you are not sure if a list item applies to your research, read the appropriate section before selecting a response.

Materials & experimental systems

n/a	Involved in the study	
☐	☒	Antibodies
☐	☒	Eukaryotic cell lines
☒	☐	Palaeontology and archaeology
☐	☒	Animals and other organisms
☒	☐	Clinical data
☒	☐	Dual use research of concern

Methods

n/a	Involved in the study	
☒	☐	ChIP-seq
☒	☐	Flow cytometry
☒	☐	MRI-based neuroimaging

Antibodies

Antibodies used	Rabbit monoclonal antibody against β-actin (ABclonal, AC038); mouse monoclonal antibodies against SNAP-25 (Synaptic systems, Cl 71.1) or Syt-1 (Synaptic systems, #105011); rabbit polyclonal antibody against SV2C (Synaptic systems, #119202). SV2 mouse monoclonal antibody (pan-SV2) was generously provided by E. Chapman (Madison, WI) and is available from Developmental Studies Hybridoma Bank (AB_2315387). Secondary antibodies were purchased from the following vendors: goat anti-rabbit-HRP (Bio-Rad, 1705046) and goat anti-mouse-HRP (Abcam, ab97023).
Validation	Each primary antibody was validated using WB for target-positive cell lysates according to manufacturer's instructions.

Eukaryotic cell lines

Policy information about cell lines and Sex and Gender in Research

Cell line source(s)	FreeStyle HEK 293 cells (R79007, ThermoFisher); HEK293T (#CRL-3216, ATCC)
Authentication	The cell lines were purchased from the manufactures, no further authentication done in the lab
Mycoplasma contamination	The cell line was tested as mycoplasma negative.
Commonly misidentified lines (See ICLAC register)	No commonly misidentified cell lines were used.

Animals and other research organisms

Policy information about studies involving animals; ARRIVE guidelines recommended for reporting animal research, and Sex and Gender in Research

Laboratory animals	Sv2a- and Sv2b- knockout mice (strain B6;129P2-Sv2atm1SudSv2btm1Sud/J, stock No: 006383; cryo recovery) were obtained from the Jackson Laboratory.<br>Mice heterozygous for both Sv2a and Sv2b were bred together to generate Sv2a+/- Sv2b-/- mice.<br>Sv2a-/-Sv2b-/- double KO pups (0 to 1-day-old) were sacrificed for neuron culture.<br>The wild-type of Sprague Dawley rat (Charles River) was used to get its embryos.<br>The MPN assay was performed employing 20–30 g swiss mice (Janvier SA, France).
Wild animals	No wild animals were used in this study.
Reporting on sex	Mouse or rat pubs were randomly sacrificed for neuron culture without sex determination.
Field-collected samples	No field-collected samples were used in this study.
Ethics oversight	All animal studies in the Dong lab were approved by the Boston Children's Hospital Institutional Animal Care and Use Committee (Protocol Number: 18-10-3794R). All procedures were approved by the Institute of Biosafety Committees at Boston Children's Hospital (Protocol Number: IBC-P00000501). The MPN assay (project license 2018/209) was performed in the Rummel lab according to §4 Abs. 3 (killing of animals for scientific purposes, German animal protection law (TSchG)). Number of animals sacrificed by trained personnel before dissection of organs were reported yearly to the animal welfare officer of the Central Animal Laboratory and to the local authority, Veterinäramt Hannover.

Note that full information on the approval of the study protocol must also be provided in the manuscript.
